# The effect of the sagittal plane osteotomy inclination on the posterior tibial slope in medial open wedge HTO: experimental study with a square column model

**DOI:** 10.1186/s12891-021-03951-0

**Published:** 2021-01-18

**Authors:** Sang Won Moon, Ji Young Ryu, Sung-Jae Lee, Sang Won Woo, Sin Hyung Park, Young Choi

**Affiliations:** 1 Department of Orthopedic Surgery, Inje University Haeundae Paik Hospital, Gimhae, South Korea; 2 Department of Occupational and Environmental Medicine, Inje University Haeundae Paik Hospital, Busan, South Korea; 3 Department of Biomedical Engineering, Inje University, Busan, South Korea; 4 Department of Orthopedic Surgery, Soonchunhyang University School of Medicine, Bucheon Hospital, Bucheon-si, Gyeonggi South Korea; 5 Department of Orthopedic Surgery, Kosin University Gospel Hospital, 262, Gamcheon-ro, Seo-gu, Busan, 49267 South Korea

**Keywords:** Tibia, Osteotomy, High tibial osteotomy, Posterior slope, Posterior tibia slope, Simulation

## Abstract

**Background:**

Medial open-wedge high tibial osteotomy (HTO) is an effective and safe treatment method for medial osteoarthritis of the knee. However, unintended changes in the posterior tibial slope (PTS) may occur. Several factors cause PTS alterations after medial open-wedge HTO; however, research on sagittal-plane osteotomy inclination (SPOI) in relation to the PTS is sparse. The purpose of this study was to evaluate whether the SPOI affects changes in the PTS after medial open-wedge HTO. The hypothesis was that an SPOI parallel to the PTS causes no change in the PTS after medial open-wedge HTO.

**Methods:**

A square column model with a 10° posterior slope was produced using two three-dimensional (3D) programs and a 3D printer. Then, a series of medial open-wedge HTO procedures was performed on the square column model through virtual simulation using the two 3D programs, and an actual simulation was conducted using a 3D printer, a testing machine and a measurement system. The SPOI was divided into four types: ① SPOI 20° (posterior-inclined 10° osteotomy), ② SPOI 10° (osteotomy parallel to posterior slope), ③ SPOI 0° (anterior-inclined 10° osteotomy), and ④ SPOI − 10° (anterior-inclined 20° osteotomy). The correction angle was increased at intervals of 5° from 0° to 30°. The change in posterior slope was measured in the sagittal plane.

**Results:**

The posterior slope was increased in SPOI 20° (posterior-inclined 10° osteotomy), maintained in SPOI 10° (osteotomy parallel to posterior slope), and decreased in SPOI 0° (anterior-inclined 10° osteotomy) and SPOI − 10° (anterior-inclined 20° osteotomy) based on the correction angle.

**Conclusions:**

In this study using a square column model, the SPOI affected the change in the PTS, and an SPOI parallel to the PTS caused no change in the PTS after medial open-wedge HTO.

## Background

Medial open-wedge high tibial osteotomy (HTO) has become an established method for medial compartment osteoarthritis of the knee with varus deformity [1–3]. Medial open-wedge HTO is a surgical method that corrects the coronal alignment of the proximal tibia, but unintended changes to the sagittal plane may also occur. One meta-analysis reported that the posterior tibial slope (PTS) increased after medial open-wedge HTO [4]. These unintended changes may influence knee kinematics and stability in the sagittal plane [5, 6].

Several studies have attempted to elucidate the cause of unintended changes in PTS after medial open-wedge HTO [2, 7–9]. Established causes include incomplete posterior cuts [7], improper gap ratios [8], and inappropriate hinge positions [2, 9]. However, investigations into the effect of the sagittal plane osteotomy inclination (SPOI) on the increase in the PTS are lacking. To the best of our knowledge, only one study has evaluated the effect of the SPOI on the PTS after medial open-wedge HTO [10].

To evaluate the exact relationship between the SPOI and PTS, an experimental study with a simplified model may represent a more efficient method compared with CT or X-ray analysis after medial open-wedge HTO. Because surgery cannot be performed perfectly in all cases, confounding variables may occur. Therefore, we thought that an experimental study is needed to control known factors affecting the PTS, such as complete posterior cut and true lateral hinge position.

The purpose of this study was to evaluate the effect of the SPOI on the change in the PTS using a square column model. We hypothesized that an SPOI parallel to the PTS would cause no change in the PTS after medial open-wedge HTO.

## Methods

**Fig. 1:**
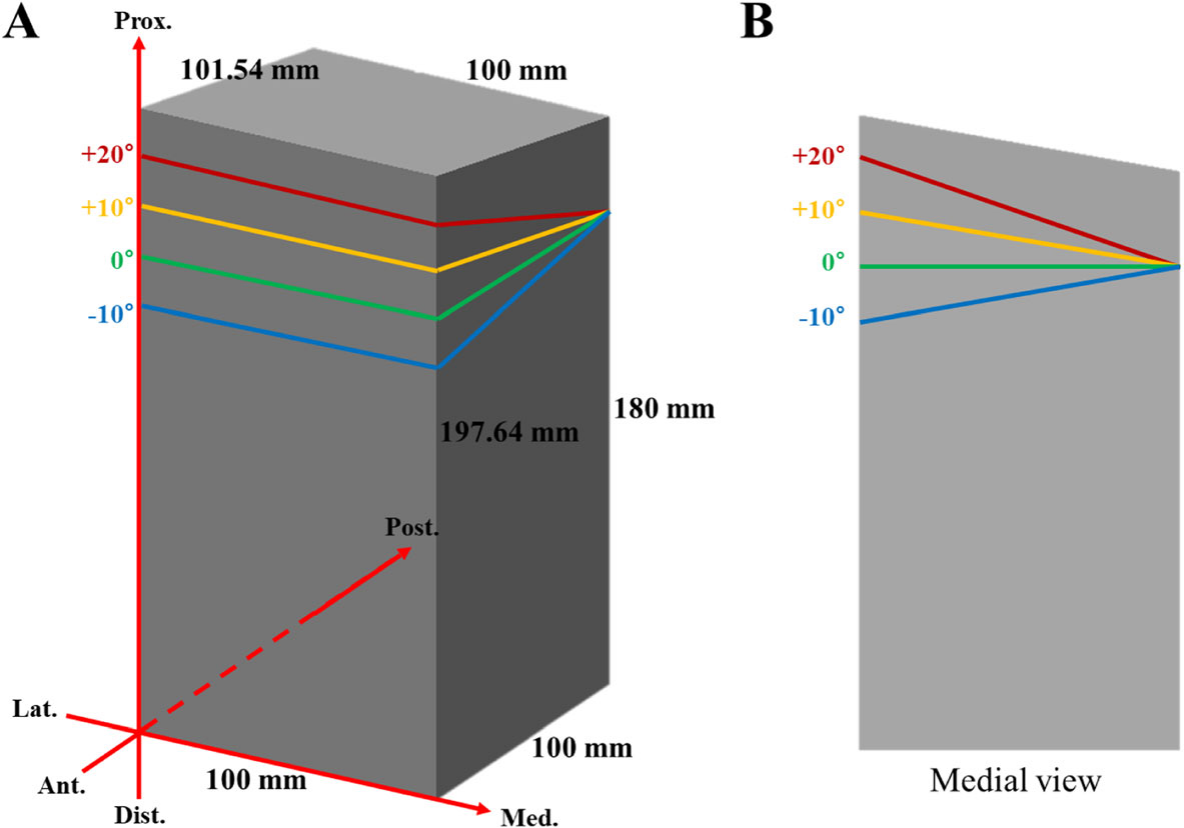
**a** A square column model with a 10° posterior slope. **b** Medial view of the square column model

To analyze the effects of the SPOI on the PTS, a square column model with a 10° posterior slope was produced (Fig. 1). A 10° posterior slope was set based on the average value previously reported [11]. Using this square column model, virtual surgery was performed, and the changes were measured.

Autodesk® Maya® software (Autodesk, San Jose, CA, USA) and Rhinoceros® software (McNeel, Seattle, WA, USA) were used for the virtual simulations. The accuracy of these software programs as tools for 3D modeling and simulation has been verified in many medical studies [12–14]. The simulation was conducted as follows. First, a virtual square column model with a 10° posterior slope was produced. Second, the SPOI was divided into four types: ① SPOI 20° (posterior-inclined 10° osteotomy), ② SPOI 10° (osteotomy parallel to posterior slope), ③ SPOI 0° (anterior-inclined 10° osteotomy), and ④ SPOI − 10° (anterior-inclined 20° osteotomy) (Fig. 1). A complete posterior cut and true lateral hinge position were applied in the simulation. A true lateral hinge position refers to a hinge position that is parallel to the osteotomy line in the axial view, and complete cutting on the square column model creates a true lateral hinge. The correction angle was increased at 5° intervals from 0° to 30°. The change in posterior slope was measured in the sagittal plane.

**Fig. 2:**
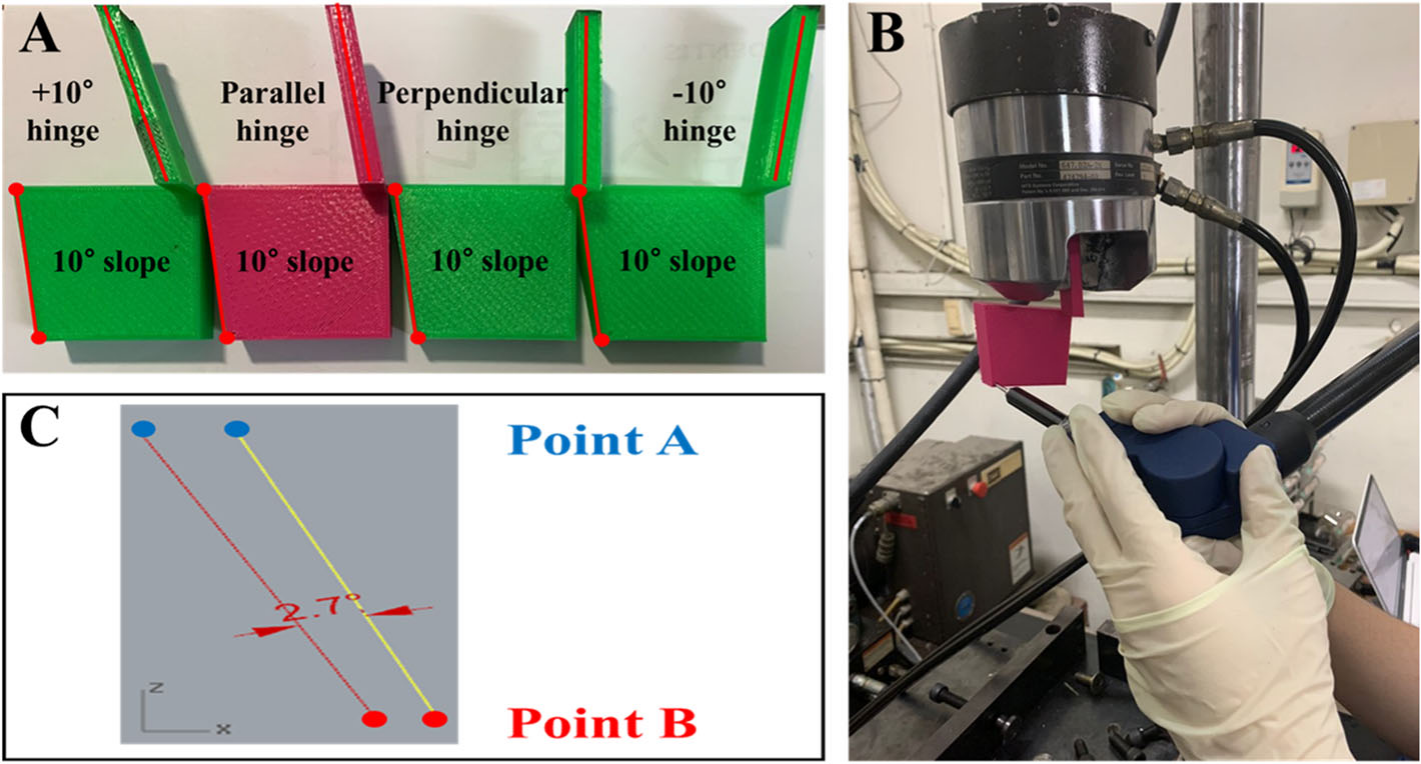
**a** Four square column models with jigs attached at different angles. **b** After correction with the MTS 858 Bionix machine (MTS System Corp), the end-point of the posterior slope was measured using a MicroScribe™ system (Revware Systems, Inc.). **c** The change in posterior slope was determined using Rhinoceros® software (McNeel)

Using a 3D printer (Objet 24, Stratasys Inc., Rehovot, Israel), four square column models, each with a 10° posterior slope, were produced. The four SPOI types were produced using jigs with different angles, which are required to attach the square column model to a testing machine (Fig. 2). The axis of coronal correction or rotation acts as a lateral hinge in medial open-wedge HTO. In the simulation using the testing machine, the axis of coronal correction or rotation was also a lateral hinge. The rotation of the square column model occurred around the lateral hinge. The central axis of the jig was the lateral hinge. Therefore, the sagittal plane angle of the jig was the SPOI.

The correction angle was increased at 5° intervals from 0° to 30° using a testing machine (MTS 858 Bionix; MTS System Corp., Eden Prairie, MN, USA) (Fig. 2). The change in posterior slope was measured 10 times using the MicroScribe™ system (Revware Systems, Inc., Raleigh, NC, USA). The reported accuracy of the device was ±0.05 mm [14]. To measure the change in posterior slope with the MicroScribe™ system, two holes were made in advance in the square column model. The anterior end-point of the posterior slope was point A and the posterior end-point of the posterior slope was point B (Fig. 2). These two points were reconstructed with a slope line using Rhinoceros® software. Using this software, the change in posterior slope was determined as the angle (Fig. 2).

Statistical analyses were performed using IBM SPSS statistics (version 25 for Windows; IBM, Armonk, NY, USA). In the actual simulation, repeated measurements of the posterior slope are presented as the means and standard deviations. Due to the small sample size and nonnormal distribution (Shapiro-Wilk test: *P* < 0.05), a nonparametric method was used as the statistical test. The differences in posterior slope by correction angle in each SPOI group were evaluated using the Kruskal-Wallis test. Statistical significance was *P* < 0.05. Additionally, the square column model in SPOI 0° (anterior-inclined 10° osteotomy) was mathematically formulated.

## Results

**Fig. 3:**
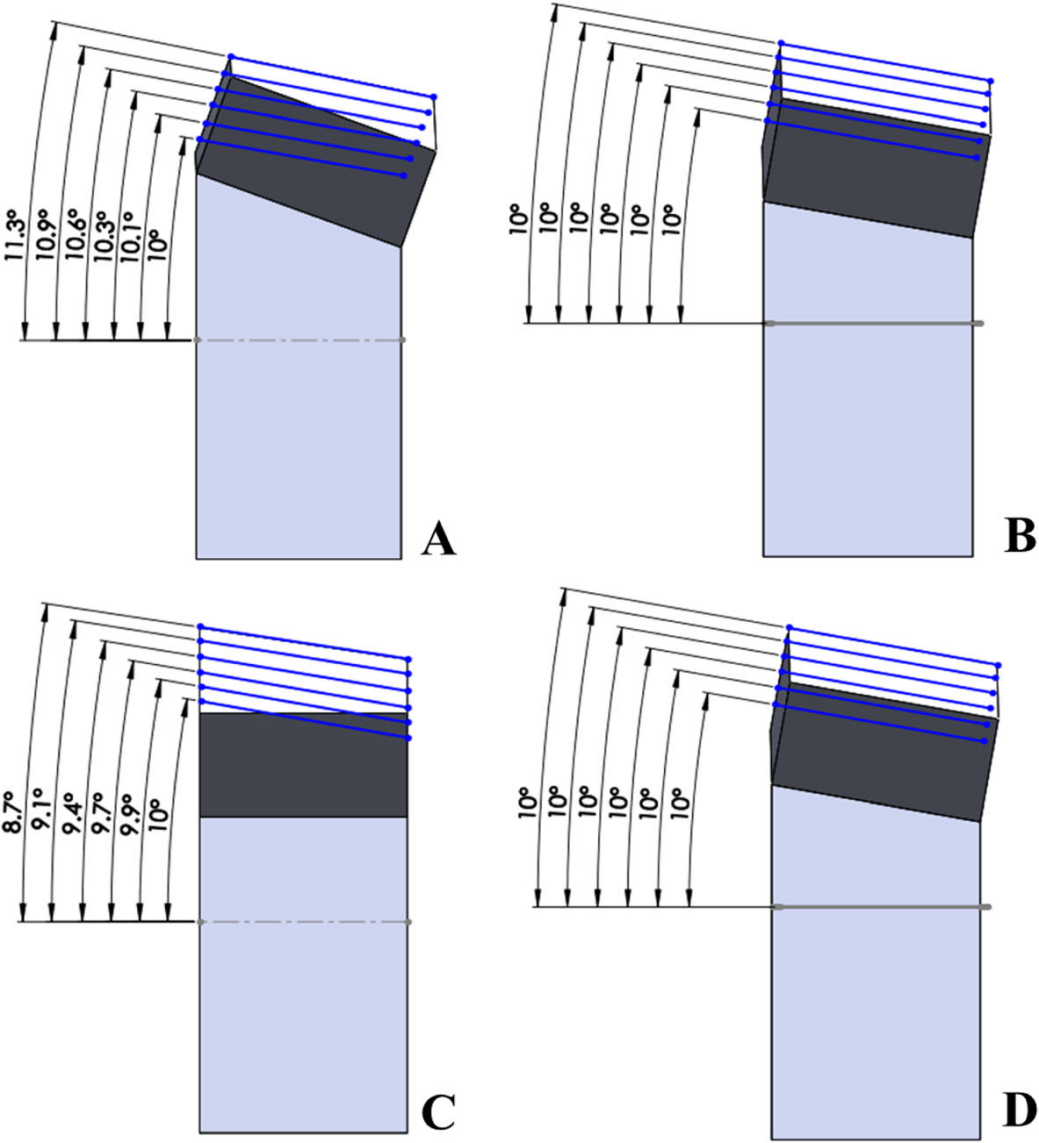
Changes in posterior slope during virtual simulation with SPOI 20° (**a**), SPOI 10° (**b**), SPOI: 0° (**c**), and SPOI -10° (**d**)

The change in posterior slope in a virtual simulation using Maya® and Rhinoceros® software is presented in Table 1. The values were the same in simulations with both programs. In SPOI 20° (posterior-inclined 10° osteotomy), the posterior slope increased with increasing correction angle from 10.0° to 11.3°. In SPOI 10° (osteotomy parallel to posterior slope), no change in posterior slope was noted based on correction angle. In SPOI 0° (anterior-inclined 10° osteotomy) and SPOI − 10° (anterior-inclined 20° osteotomy), the posterior slope decreased based on the correction angle, and a greater decrease was noted for SPOI − 10° compared with SPOI 0° (Table 1) (Fig. 3).

**Table 1:** Changes in posterior slope during virtual simulation. (Maya® and Rhinoceros® software)

		SPOI 20°	SPOI 10°	SPOI 0°	SPOI −10°
Correction angle	5°	10°	10°	10°	9.9°
	10°	10.1°	10°	9.9°	9.7°
	15°	10.3°	10°	9.7°	9.4°
	20°	10.6°	10°	9.4°	8.9°
	25°	10.9°	10°	9.1°	8.3°
	30°	11.3°	10°	8.7°	7.5°

**Fig. 4:**
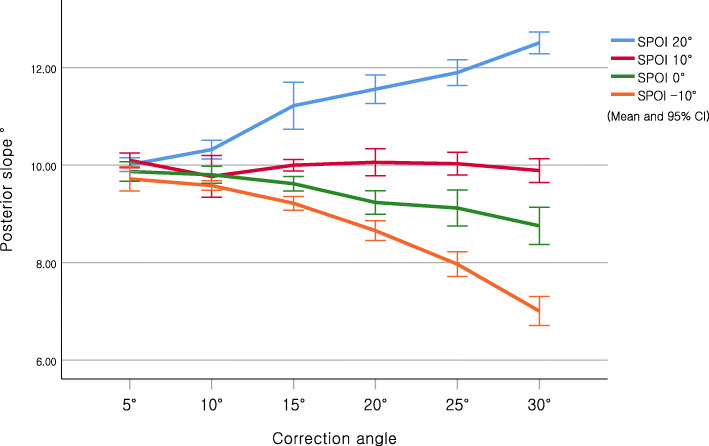
Changes in posterior slope based on sagittal plane osteotomy inclination (SPOI) in the actual simulation

Table 2 shows the change in posterior slope in an actual simulation using a 3D printer. As noted in the virtual simulation, the posterior slope was significantly decreased with increasing correction angle in anterior-inclined 10° and 20° osteotomies (SPOI 0° and − 10°) (*P* < 0.001). In contrast, the posterior slope was significantly increased in posterior-inclined 10° osteotomy (SPOI 20°) (P < 0.001). No significant difference in the posterior slope with increasing correction angle in osteotomy parallel to the posterior slope (SPOI 0°) was noted (*p* = 0.485). Figure 4 presents the means and 95% confidence intervals of the posterior slope according to the SPOI groups.

**Table 2:** Mean and standard deviation of posterior slope according to sagittal plane osteotomy inclination (SPOI) in the actual simulation (*n* = 10)

		SPOI 20°	SPOI 10°	SPOI 0°	SPOI −10°	*P*-value^a^
Correction Angle	5°	10.0 ± 0.2°	10.1 ± 0.2°	9.9 ± 0.3°	9.7 ± 0.3°	0.016
	10°	10.3 ± 0.3°	9.8 ± 0.6°	9.8 ± 0.2°	9.6 ± 0.1°	< 0.001
	15°	11.2 ± 0.7°	10.0 ± 0.2°	9.6 ± 0.2°	9.2 ± 0.2°	< 0.001
	20°	11.6 ± 0.4°	10.1 ± 0.4°	9.2 ± 0.3°	8.7 ± 0.3°	< 0.001
	25°	11.9 ± 0.4°	10.0 ± 0.3°	9.1 ± 0.5°	8.0 ± 0.4°	< 0.001
	30°	12.5 ± 0.3°	9.9 ± 0.3°	8.8 ± 0.5°	7.0 ± 0.4°	< 0.001
*P*-value^a^		< 0.001	0.485	< 0.001	< 0.001	

^a^Kruskal-Wallis test

**Fig. 5:**
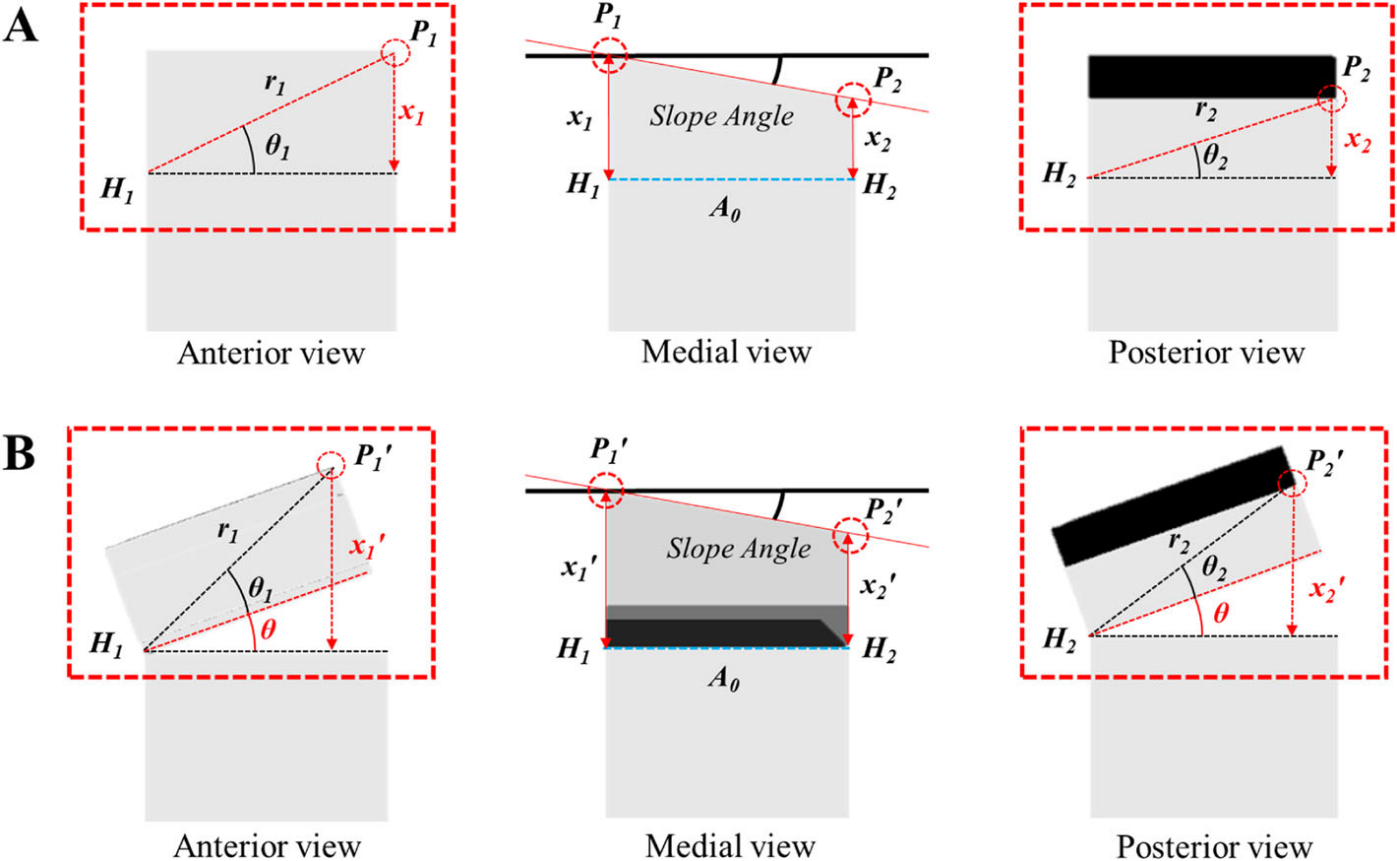
Mathematic modeling of SPOI 0° (anterior-inclined 10° osteotomy). **a** Before correction; **b** After correction; P1: anterior end point; P2: posterior end point; H1: anterior end point of the lateral hinge; H2: posterior end point of the lateral hinge; r1: distance from H1 to P1; r2: distance from H2 to P2; θ1: angle of P1 with the OP line; θ2: angle of P2 with the OP line; ϰ1=*r*1xsin(θ1); ϰ2=*r*2xsin(θ2); ϰ1′=*r*1xsin(θ1 + θ); ϰ2′=*r*2xsin(θ2 + θ)

Mathematical modeling was applied in SPOI 0° (anterior-inclined 10° osteotomy) (Fig. 5). If θ1 and θ2 are the same value, the change from × 1 and × 2 to × 1’ and × 2’, respectively, is the same. Therefore, the posterior slope is the same. If θ1 and θ2 are different values, the resulting values are different, and the posterior slope is therefore altered. Thus, for θ1 and θ2 to be the same value, the SPOI should be parallel to the posterior slope.

## Discussion

The present study showed that the SPOI affected the posterior slope in a square column model. The posterior slope was maintained in SPOI 10° (osteotomy parallel to the posterior slope), increased in SPOI 20° (posterior-inclined 10° osteotomy), and decreased in both SPOI 0° (anterior-inclined 10° osteotomy) and SPOI − 10° (anterior-inclined 20° osteotomy) in virtual simulation. Additionally, the same results were shown with statistical significance in the actual simulation.

Previous studies have recommended that the osteotomy line in the sagittal plane needs to be parallel to the PTS in medial open-wedge HTO [15–17]. Miller et al. [18] stated that they maintained an osteotomy line parallel to the PTS in medial open-wedge HTO to avoid inadvertent alterations of the native PTS. Amendola et al. [19] suggested that a parallel osteotomy line is needed because osteotomy perpendicular to the tibial sagittal axis would create a very thin bony fragment posteriorly. However, the scientific evidence supporting the use of a parallel osteotomy line remains inadequate.

Lee et al. [10] reported that only 12.9% of patients underwent parallel osteotomy, and 87.1% of patients underwent anterior-inclined osteotomy despite the surgeon attempting to perform parallel osteotomy. In this study, changes in the PTS after surgery were significantly correlated with sagittal osteotomy inclination. Although the results for parallel osteotomy are consistent with our findings, a difference in the direction of posterior slope change is noted. Our results showed that the posterior slope was decreased in anterior-inclined 10° and 20° osteotomies (SPOI 0° and − 10°). Lee et al. [10] reported that the PTS was increased in the anterior-inclined osteotomy group. This discrepancy may have resulted from the difference in hinge position. The anterior-inclined osteotomy group may have gained an increased PTS due to the posterolateral hinge position.

Akamatsu et al. [20] reported no significant difference between the sagittal osteotomy plane angle and the change in the PTS, whereas anteroposterior hinge position ratio was significantly correlated with the change in the PTS. These results were inconsistent with our findings. However, these authors could not control for the effect of hinge position in their analysis of the association between the sagittal osteotomy plane angle and changes in the PTS. We suggest that hinge position must be considered when evaluating the effect of the SPOI on changes in the PTS.

This study had some limitations that need to be considered. First, it was an experimental study performed using a square column model with two 3D programs and a 3D printer. However, although the complex structure of the proximal tibia was simplified to a square column model, the relationship between the variables we sought to observe was not compromised. Rather, it was possible to analyze the relationship between the SPOI and the PTS accurately without confounding factors. However, due to the difference in structure, there may be a difference in the amount of change observed. Therefore, in the proximal tibia, the amount of change in the PTS may be greater than that observed in this experiment. Second, this study did not consider the soft tissue around the knee, which could affect the results. Third, the reported differences in the PTS may not seem clinically relevant. However, the change in the PTS was evaluated only in the lateral hinge position. If the SPOI is increased in the posterolateral hinge position, the change in PTS may be much greater. Therefore, further model studies are needed to demonstrate the effect of the SPOI on changes in the PTS according to hinge position. Fourth, virtual simulation results and actual simulation results did not match perfectly. The actual simulation was performed using the MTS 858 Bionix testing machine and the MicroScribe™ measurement system. Therefore, the measurement error of the actual simulation may be greater than that of the virtual simulation.

Although there were several limitations, this square column model study has the advantage of completely controlling other factors affecting the PTS during measurement. Thus, our study shows the relationship between changes in the PTS and the SPOI, excluding all other factors related to the PTS.

## Conclusions

In this study using a square column model, the SPOI affected the change in the PTS, and an SPOI parallel to the PTS caused no change in the PTS after medial open-wedge HTO.

## Data Availability

All data generated or analyzed during this study are included in this published article.

## Abbreviations

HTO: High tibial osteotomy
PTS: Posterior tibial slope
SPOI: Sagittal plane osteotomy inclination
