# Correction to: The effect of the sagittal plane osteotomy inclination on the posterior tibial slope in medial open wedge HTO: experimental study with a square column model

**DOI:** 10.1186/s12891-021-04169-w

**Published:** 2021-03-23

**Authors:** Sang Won Moon, Ji Young Ryu, Sung-Jae Lee, Sang Won Woo, Sin Hyung Park, Young Choi

**Affiliations:** 1 Department of Orthopedic Surgery, Inje University Haeundae Paik Hospital, Gimhae, South Korea; 2 Department of Occupational and Environmental Medicine, Inje University Haeundae Paik Hospital, Busan, South Korea; 3 Department of Biomedical Engineering, Inje University, Busan, South Korea; 4 Department of Orthopedic Surgery, Soonchunhyang University School of Medicine, Bucheon Hospital, Bucheon-si, Gyeonggi South Korea; 5 Department of Orthopedic Surgery, Kosin University Gospel Hospital, 262, Gamcheon-ro, Seo-gu, Busan, 49267 South Korea

**Correction to: BMC Musculoskelet Disord 22, 89 (2021)**

**https://doi.org/10.1186/s12891-021-03951-0**

Following the publication of the original article [1] the authors found that panels b and d in Fig. 3 have the same image. The original article [1] has been updated.

The correct Fig. 3 is shown below.

**Fig. 3:**
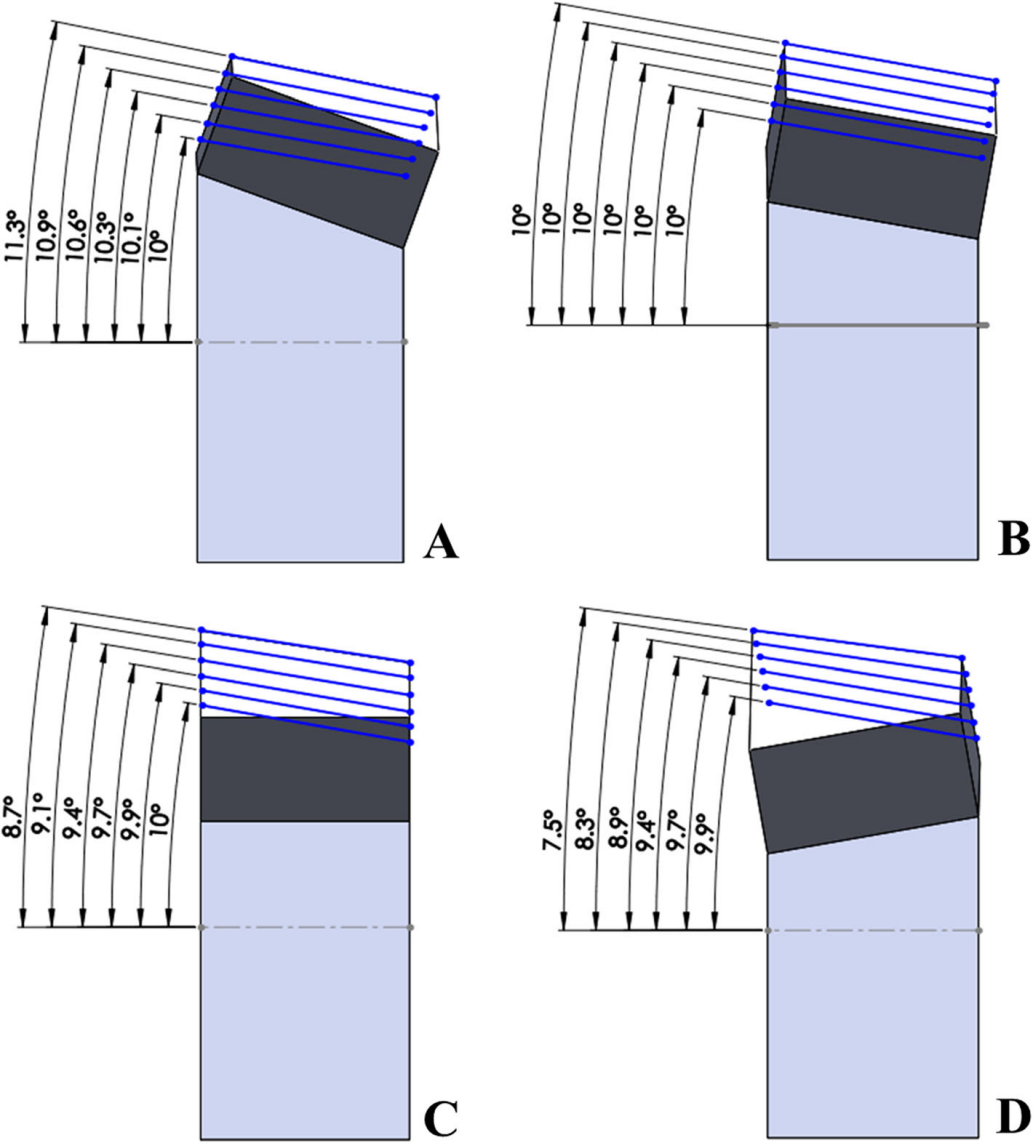
Changes in posterior slope during virtual simulation with SPOI 20° (**a**), SPOI 10° (**b**), SPOI: 0° (**c**), and SPOI -10° (**d**)
